# Protocol for a feasibility randomized controlled trial to evaluate the efficacy, safety and tolerability of N-acetylcysteine in reducing adverse drug reactions among adults treated for multidrug-resistant tuberculosis in Tanzania

**DOI:** 10.1186/s40814-023-01281-7

**Published:** 2023-04-01

**Authors:** Stellah G. Mpagama, Happiness C. Mvungi, Peter M. Mbelele, Hadija H. Semvua, Alphonce A. Liyoyo, Kristen Petros de Guex, Derek Sloan, Gibson S. Kibiki, Martin Boeree, Patrick P. J. Phillips, Scott K. Heysell

**Affiliations:** 1 Kibong’oto Infectious Diseases Hospital–Sanya Juu Siha/Kilimanjaro Clinical Research Institute, Mae Street, Lomakaa Road, Siha Kilimanjaro, Tanzania; 2 Kilimanjaro Christian Medical University College, Moshi, Kilimanjaro Tanzania; 3 Nelson Mandela African Institute of Science and Technology, Arusha, Tanzania; 4 Division of Infectious Diseases and International Health, University of Virginia, Charlottesville, USA; 5 University of St. Andrews, St. Andrews, UK; 6 Africa Research Excellence Fund, London, UK; 7 Radboud Institute for Health Sciences, Radboud University Medical Center, Nijmegen, the Netherlands; 8 UCSF Center for Tuberculosis, University of California San Francisco, San Francisco, USA

**Keywords:** Multidrug-resistant tuberculosis, Adverse drug reactions, N-acetylcyteine, Drug-induced Liver injury, Clinical trial

## Abstract

**Background:**

Adverse drug reactions (ADRs) frequently occur in patients using second-line anti-tuberculosis medicine for treatment of multidrug resistant tuberculosis (MDR-TB). ADRs contribute to treatment interruptions which can compromise treatment response and risk acquired drug resistance to critical newer drugs such as bedaquiline, while severe ADRs carry considerable morbidity and mortality. N-acetylcysteine (NAC) has shown promise in reducing ADRs for medications related to TB in case series or randomized controlled trials in other medical conditions, yet evidence is lacking in MDR-TB patients. TB endemic settings have limited capacity to conduct clinical trials. We designed a proof-of-concept clinical trial primarily to explore the preliminary evidence on the protective effect of NAC among people treated for MDR-TB with second-line anti-TB medications.

**Methods:**

This is a proof-of-concept randomized open label clinical trial with 3 treatment arms including a control arm, an interventional arm of NAC 900 mg daily, and an interventional arm of NAC 900 mg twice-daily administered during the intensive phase of MDR-TB treatment. Patients initiating MDR-TB treatment will be enrolled at Kibong’oto National Center of Excellence for MDR-TB in the Kilimanjaro region of Tanzania. The minimum anticipated sample size is 66; with 22 participants in each arm. ADR monitoring will be performed at baseline and daily follow-up over 24 weeks including blood and urine specimen collection for hepatic and renal function and electrolyte abnormalities, and electrocardiogram. Sputum will be collected at baseline and monthly thereafter and cultured for mycobacteria as well as assayed for other molecular targets of *Mycobacterium tuberculosis*. Adverse drug events will be analysed over time using mixed effect models. Mean differences between arms in change of the ADRs from baseline (with 95% confidence intervals) will be derived from the fitted model.

**Discussion:**

Given that NAC promotes synthesis of glutathione, an intracellular antioxidant that combats the impact of oxidative stress, it may protect against medication induced oxidative damage in organs such as liver, pancreas, kidney, and cells of the immune system. This randomized controlled trial will determine if NAC leads to fewer ADRs, and if this protection is dose dependent. Fewer ADRs among patients treated with MDR-TB may significantly improve treatment outcomes for multidrug regimens that necessitate prolonged treatment durations. Conduct of this trial will set the needed infrastructure for clinical trials.

**Trial registration:**

PACTR202007736854169 Registered 03 July 2020

**Supplementary Information:**

The online version contains supplementary material available at 10.1186/s40814-023-01281-7.

## Key questions on feasibility

- Tanzania is one of the sub-Saharan African countries facing a high burden of tuberculosis (TB) including the multidrug resistant (MDR)-TB and other infectious diseases, however the capacity to conduct research, particularly clinical trials, has been limited. We proposed to conduct a clinical trial among MDR-TB patients with the mains components of toxicity reduction and capacity development in clinical trials in Tanzania.
- To our knowledge, this is the first registered global trial to examine the protective effect of N-acetylcysteine (NAC) on occurrence of adverse drug reaction in MDR-TB patients treated with current recommended regimens that prioritize all-oral medications.

What uncertainties existed regarding feasibility?

- It was uncertain whether Tanzanian institutes would execute both sponsor/investigator responsibilities and trial management roles to address clinical research questions especially in one of the priority infectious diseases, MDR-TB.
- It was also uncertain whether a clinical trial unit established with managers, physicians, trialists and laboratory scientists would be set to execute all relevant clinical trials processes and confront infectious diseases using MDR-TB as a model disease.

What are the expected implications of the feasibility findings for the main study?

- This clinical trial aimed to minimize occurrence of adverse drug reactions in MDR-TB patients that would have a direct patient benefit but with additional components of the trial capacity for laboratory scientists, research trialists in order to create a rapid response infrastructure for other infectious diseases of public health priority. Furthermore, the trial will explore the preliminary evidence on the effect of NAC on the results that will guide design of the pragmatic clinical trial in Tanzania.

## Introduction

### Background and rationale

The crisis of multidrug-resistant tuberculosis (MDR-TB), defined as point mutations of *Mycobacterium tuberculosis* (MTB) chromosomes resulting in strains that are resistance to isoniazid and rifampicin, key medicines for the treatment of TB, continues to worsen [1]. In 2019, the World Health Organization (WHO) estimated 484,000 cases of MDR-TB, while approximately 156,000 were enrolled to initiate treatment. This marked a 21% increase in enrolment compared to the 2016 report [2]. With the growing epidemic of MDR-TB, further exacerbated by the COVID-19 pandemic [3], and a greater proportion of people accessing treatment, there is further urgency to assure treatment regimens are tolerable and completed without interruption which poses risk to individual outcomes and *M. tuberculosis* strains with amplified drug resistance [4]. Despite the priority for tolerable regimens, medicines used to treat MDR-TB are referred to as “second-line” because of reduced potency and a worse side effect profile [5]. Regimens are constructed of 4–6 drugs based on the susceptibility profile of the infecting *M. tuberculosis* strain, drug-drug interactions or individual contraindications, and supply chain availability [6]. New and repurposed drugs have improved the efficacy and shortened the duration of MDR-TB treatment but those medications have significant and often overlapping toxicities [7]. Based on data from controlled trials and large operational studies, the WHO has recently prioritized bedaquiline, linezolid, newer generation fluoroquinolones (moxifloxacin or levofloxacin), and clofazimine for the treatment of MDR-TB as well as consideration of other new agents such as delamanid and conventional anti-TB drugs such as cycloserine, pyrazinamide, ethionamide/prothionamide, and ethambutol to complete a multi-drug regimen [8]. While medications requiring intravenous or intramuscular injection are no longer preferred, there are some uncommon clinical scenarios where TB treatment programs or patients themselves may opt to include an injectable aminoglycoside (amikacin or streptomycin) or a carbapenem with beta-lactamase inhibitor (meropenem/clavulanate or imipenem-cilastin/clavulanate).

No other infectious disease requires treatment strategies of such quantity of drugs, and drug class diversity as MDR-TB, and combined toxicities are not dissimilar to treatment of hematological malignancy. As described elsewhere, the severe adverse drug reactions (ADRs) from medications used to treat MDR-TB include hepatotoxicity, cytopenias, QTc prolongation and cardiac arrhythmia, nephrotoxicity, ototoxicity, peripheral and optic neuropathy, and psychosis [9, 10]. Given the combination of drug-classes, including broad spectrum classes such as the fluoroquinolones, gastrointestinal side effects are common, and can even confuse the presentation of more serious ADRs including hepatoxicity and lactic acidosis [11]. For example, despite the efficacy of bedaquiline, up to 20.8% of people experience bedaquiline related ADRs, with 7.4% serious. ADRs related to bedaquiline were most frequently gastrointestinal (14%), followed by metabolic disorders (8.5%) and nervous system disorders (8.5%) [12]. Likewise, the inclusion of linezolid in MDR-TB regimen has a significant positive effect in improving treatment outcome and reducing mortality, yet the occurrence of ADRs include myelosuppression (33%), neuropathy (30%) whereas other less common ADRs are vomiting, hyperpigmentation and transient visual impairment [13]. Clofazimine is considered safe since the ADRs requiring discontinuation or withdrawal reported have been as low as 0.1% common ADRs are skin discolouration and gastrointestinal side effects with a pooled proportion of 22% [14]. Furthermore, several drugs have overlapping ADRs for instance the fluoroquinolones, bedaquiline, and delamanid all may cause QTc interval prolongation and hepatotoxicity [15].

We have designed the following trial to study a promising compound, N-acetylcysteine (NAC), for reduction of ADRs during the course of MDR-TB treatment, which is to our knowledge the first of its kind with this primary objective for MDR-TB. NAC is a thiol compound and the acetylated form of L-cysteine with the chemical formula C5H9NO3S and molecular weight of 163.2 g/mol. NAC is efficiently absorbed and metabolized primarily by the liver. Absorption provides a large amount of NAC for cellular uptake, deacetylation of cysteine, and synthesis of glutathione (GSH). GSH is an intracellular antioxidant that combats the impact of oxidative stress thus protecting the vital cellular components against the dangerous effect of peroxidation. The free sulfyhydryl group in GSH readily scavenges harmful radicals such as reactive oxygen species, peroxides and superoxides to thiyl radical, which rapidly dimerises to form glutathione disulfide [16, 17]. GHS released from NAC then carriers out its putative protective effect both enzymatically and non-enzymatically. The benefit of NAC on oxidative damage extend to organs such liver, pancreas, kidney, inner ear hair cells, and cells of the immune system. Furthermore, NAC demonstrates anti-inflammatory properties by limiting pro-inflammatory cytokine release, particularly through a NF-kappa beta pathway [18].

NAC has been employed in clinical practice for several decades. It has been used as a mucolytic agent and for the treatment of numerous disorders including paracetamol intoxication, doxorubicin cardiotoxicity, ischemia–reperfusion cardiac injury, acute respiratory distress syndrome, bronchitis, chemotherapy-induced toxicity, heavy metal toxicity and psychiatric disorders [19]. Most relevant to prevention of ADRs from MDR-TB treatment, previous NAC interventions have been trialed in drug susceptible (DS)-TB among primarily Asian populations [20, 21]. Baniasadi et al. conducted a clinical trial in an older age (≥ 60 years) population at higher risk of drug induced liver injury (DILI). The hepatoprotective effect of NAC was significant since anti-TB DILI occurred in 12 patients (37.5%) in the control group but none in the NAC group [20]. Farazi et al. conducted another NAC trial in 85 patients with age ≥ 50 years and treated for DS-TB [21]. Eligible participants were randomly selected to receive NAC 600 mg or placebo with standard rifampicin, isoniazid, pyrazinamide and ethambutol (RHZE) treatment. DILI occurred in 14.3% of the placebo group as signified by raised serum aspartate transaminases (AST), alanine transaminases (ALT) and bilirubin. Interestingly, the group receiving NAC also had reduced levels of AST, ALT and bilirubin compared to their baseline values [21]. A further meta-analysis by Kranzer et al. conducted a review on the efficacy and safety of NAC in preventing aminoglycoside induced ototoxicity and found the weight of evidence supporting the safety and otoprotective effect of NAC when co-administered with an aminoglycoside, even for durations shorter than those used for MDR-TB [22]. Thus, the prior studies in non-MDR-TB populations and among people with drug-susceptible TB provide a strong justification for a clinical trial to investigate the effect of concomitant NAC treatment in patients receiving MDR-TB treatment. If NAC allows uninterrupted use of the most efficacious dose and duration while limiting the long-term sequela of MDR-TB treatment, then the trial may indeed contribute to the current ambitious goals set by the global health community for achieving a 90% TB treatment success rate by 2035 [23]. Hence, conducting this randomized controlled trial within a MDR-TB program from a TB endemic country will provide actionable programmatic data with which to determine the eventual role of a NAC intervention.

## Clinical trial objectives

### Hypothesis I

Administration of oral NAC at a dose of 900 mg daily in combination with second-line during treatment of multidrug-resistant tuberculosis (MDR-TB) will protect against occurrence of serious advent events without interfering with the effect of second line anti-TB regimen.

### Hypothesis II

Administration of oral NAC at a dose of 900 mg twice a day in combination with second-line during treatment of multidrug resistant tuberculosis (MDR-TB) will protect more than the dose of 900 mg daily against occurrence of serious adverse events without interfering with the effect of second line anti-TB regimen.

### Hypothesis III

Hands-on training, capacity development and setting infrastructure for clinical trials will provide a comprehensive foundation for addressing TB and other infectious diseases of priority in Tanzania.

## Primary objective

To explore the preliminary evidence on the protective effect of NAC through assessing the development of clinical or laboratory-based adverse drug reactions (ADRs) at any frequency during the first six months of local standard of care MDR-TB treatment among patients receiving NAC at a dose of 900 mg daily or 900 mg twice a day compared to those patients treated with the standard of care regimen and placebo.

## Trial pilot and feasibility objectives

To establish the clinical trial unit equipped with standards of operations, clinical trialists, laboratory scientists, and mid-level personnel and create a rapid response infrastructure that will address TB clinical research questions and other infectious diseases in Tanzania.

## Trial design

NAC trial is a phase 2b randomized open labelled superiority trial with three parallel groups and a primary endpoint of occurrence of ADRs at any time during treatment for MDR-TB.

## Methods: participants, interventions, and outcomes

### Study setting

Patients will be recruited at one center, Kibong’oto Infectious Diseases Hospital (KIDH) in Tanzania. The site is experienced in recruitment, hospitalization, safety and efficacy measurement and has the capacity to receive more than 100 MDR-TB patients per year [24]. KIDH has also participated in the International Collaboration for Infectious Diseases Research (ICIDR) consortium to build capacity for MDR-TB trials recruitment and follow-up of a longitudinal cohort of MDR-TB participants through 96 weeks (NCT 03,559,582). In addition, the KIDH research team has demonstrated the capacity of recruiting DS-TB patients for other trials in the Pan African Consortium for Evaluating Anti-TB Antibiotics (PanACEA) with a remarkably high proportion of retention [25].

KIDH is one of the sites determined to set the clinical trial infrastructure for addressing infectious diseases clinical challenges. Currently, the KIDH is developing personnel particularly the clinical trialists, laboratory scientists, and other intermediate staff for conducting clinical trial. The site has demonstrated an initial research capacity through conducting a longitudinal cohort of MDR-TB as a mechanism to prepare for clinical trial [26]. Ongoing research capacity strengthening model at KIDH includes academic training Master or PhD or postdoctoral training, mentoring, collaborations, and networking.

### Eligibility criteria

MDR-TB participants will be eligible, if they are able and willing to provide a written informed consent prior to participation, with age range between 18 and 65 years. They should be newly diagnosed with MDR-TB without a history of using or being on MDR-TB treatment. Decisions on composition of the MDR-TB treatment regimen and timing of initiation will be made by the treating clinicians, but study participants will only be eligible if Karnofsky score of ≥ 50 defined as individuals requiring less considerable or frequent medical care. Female participants should not be pregnant as confirmed by urinary pregnant test to ensure homogeneity of the participants.

Other exclusion criteria include people with previous existing pathology which will preclude testing such as severe baseline hearing loss, central nervous system pathology (i.e., major head trauma, meningitis, encephalitis, or brain metastasis), or untreated mood disorders such as schizophrenia, schizoaffective disorder or psychotic disorder, or using psychotherapeutics like imipramine, and escitalopram. Similarly, participants with known comorbid conditions such as severe liver or renal diseases will be excluded.

### Who will take informed consent?

Recruitment will take place among individuals to KIDH for MDR-TB treatment. Upon identification of a potentially eligible participant, study staff (research nurse or research doctor) will provide information about the study to the participant. As described in greater detail in the study-specific standard operating procedure, the informed consent process will include detailed review of the study informed consent form (ICF) and will allow time to address any questions or concerns each participant may have, and an assessment of each participant’s understanding will be performed before proceeding to the informed consent decision. Illiterate adult patients will have to thumbprint the consent sheet in presence of an impartial witness who will sign the consent sheet in addition. The process will be fully documented and only participants who are able to demonstrate understanding will be asked to provide written informed consent to take part in the study. Written informed consent for study participation must be obtained before any study related procedures are performed. Screening evaluations must be performed within 7 days of entry. Participants screening and enrolment registers will be used to assist with tracking the screening and enrolment process. When informed consent is obtained for the study, a participant identification screening (PID) number will be assigned and eligible participants will receive enrolment PID number. For participants who are found to be ineligible for the study, or who do not enrol in the study for any reason, an electronic case report form (eCRF) will be completed to record the screening outcome.

### Additional consent provisions for collection and use of participant data and biological specimens

Additional consent will be administered for phlebotomy for full pharmacokinetic sampling, which will be performed at 2 weeks after initiation of treatment. The consent will cover week 2 venous blood collection that will be drawn at 1, 2, 6, and 10–12 (late sample per site feasibility) hours and week 8 drawn at 2 and 6 h after medication administration with attempt by single venipuncture for peripheral IV insertion. Such sampling allows for adequate calculation of serum peak (*C*_max_) and estimate total exposure (AUC) of the MDR-TB drugs in the regimen to determine if NAC co-administration unexpectedly impacts these parameters. A maximum of 7 ml will be obtained in heparinized tubes at each draw as up to 4 drugs will be required to be assayed. Additional consent will be obtained for other baseline specimens like stool, urine and saliva for pre-planned future studies.

### Interventions

We will use n-acetylcysteine (NAC) 900 mg in the effervescent tablet preparation as study drug. NAC is manufactured by BioAdventex Pharma Inc., Canada, based on good manufacturing practice standards. One intervention group will receive NAC at a dose of 1800 mg divided twice daily with second line MDR-TB drugs and another intervention group will receive NAC at a dose of 900 mg second line MDR-TB drugs. The control group will receive only second line MDR-TB drugs.

Participants will stop the use of trial medications if there will be any serious adverse events related to the use of NAC for instance allergic reactions or if a participant opts to withdraw from continuing with research participation.

Study nurses will be responsible for observing the participants taking the investigational medicinal product (IMP) during the treatment phase. Study nurses will provide the participants daily IMP and will document administration in the IMP treatment adherence chart. The site trial pharmacist/delegated dispenser will be responsible for dispensing the IMP. Accurate accountability records will be kept by the site to assure that the IMP will not be dispensed to any person who is not a subject under the terms and conditions set forth in this protocol, i.e., delivery to site, inventory at site, use by subject, destruction etc. The investigator/designee will immediately inform the sponsor of any quality issues arising with respect to the IMP. The sponsor will take whatever action is required should such a situation arise. The investigator undertakes to use the IMP only as indicated in this protocol.

### Relevant concomitant care permitted or prohibited during the trial

NAC interacts with nitroglycerin resulting in formation of S-nitroso-NAC, which strongly inhibit platelet aggregation whereas the free sulfhydryl donated from NAC can potentiate the systemic, and coronary vasodilator effects of nitroglycerin in patients with acute myocardial infarction or angina pectoris [27, 28]. This effect increases the risk of hypotension. Likewise, the sulfhydryl NAC modifies the renin-angiotensin II, possibly by inhibition of angiotensin converting enzyme inhibitors, thus reduces conversion of angiotensin I to angiotensin II [29, 30]. Through different mechanisms, NAC modulates glutamate and may results in clinically relevant psychopharmacological properties. In an animal model, Costa-Campos et al. investigated the combination of NAC with antidepressant drugs [31]. Findings show NAC reduced the potency of imipramine and escitalopram but not those of desipramine and bupropion. Conversely, in the same model NAC potentiates fluoxetine. Therefore, participants using imipramine and escitalopram will be excluded while fluoxetine may require dose adjustment.

Although there is no concrete evidence on the interaction of NAC with second line anti-TB drugs for MDR-TB, a number of antimicrobial drugs have the potential to exert central nervous system (CNS) effects and many are associated with stimulant, psychotomimetic and epileptogenic properties [32]. For example, fluoroquinolones can cause CNS effects mediated by gamma-aminobutyric acid (GABA) antagonism, cycloserine and aminoglycosides use can lead to N-Methyl-D-Aspartate (NMDA) agonism, and linezolid and isoniazid can exert monoamine oxidase (MAO) inhibition. In this trial, participants that will be using the psychotomimetic or epileptogenic agents, they will receive second line anti-TB medication without dose adjustment; however, they will be monitored closely according to the protocol.

## Outcomes

### Primary endpoint

The primary outcomes of interests include feasibility metrics related to implementation of sponsor/investigator responsibilities and trial management roles, knowledgeable and skilled trial manager, physician trialists, and laboratory scientists. Evidence generated through this process will guide on the design of the pragmatic trial of NAC in MDR-TB patients.

### Secondary end-point is based on safety and tolerability

Patients will be daily assessed for adverse events including vital signs, physical examinations, routine clinical laboratory tests such as complete metabolic panel, complete blood count, and urinalysis, and interval assessment of electrocardiogram and for those using injectable agents, audiometry testing.

- Proportion of adverse events
- Proportion of adverse events related to experimental treatment

### Secondary endpoints

#### Efficacy

- Proportion of MDR-TB patients requiring second-line anti-TB regimen modifications due to ADRs
- Time for development of AE including the SADRs
- TB Symptoms profile (Appendix 1)
- Time to first negative culture on solid/liquid media
- Proportion of patients converting to negative sputum culture on solid culture at 4, 6, or 8 months after treatment initiation
- Rate of change of sputum MTB RNA in MBL assay during treatment

#### Pharmacokinetics endpoints of second-line anti-TB drugs

- Area under the curve (AUC)
- Observed *C*_max_ and *T*_max_
- Volume of distribution
- Elimination half life

### Participant timeline

**Fig. 1:**
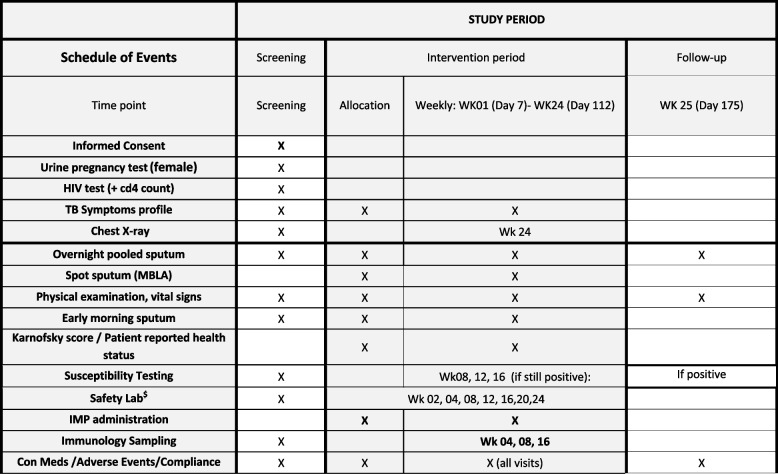
Schedule of events WK denotes week $ The safety parameters includes Hematology: hemoglobin, hematocrit, red blood cell count, white blood cell count with differential, platelet count. Clinical Chemistry: Albumin, urea, creatinine, direct, indirect and total bilirubin, uric acid, total protein, ALP, AST, ALT, gamma‐glutamyl transferase (GGT), lactate dehydrogenase (LDH), phosphate, sodium, potassium, calcium (corrected for albumin), chloride, random/fasting glucose, bicarbonate/CO2

Screening may be initiated after a written informed consent is obtained. Screening procedures will be performed on multiple days, including on the date of enrolment. For potential participants who do not meet the eligibility criteria, screening may be discontinued once ineligibility is determined. The information will be entered in the case record form to record the screening outcome. Subjects, who following the screening assessments are eligible for the trial and willing to participate, will be randomized/enrolled into the trial and assigned a randomization number. Participant will be followed according to the schedule of events as described in Fig. 1

### Sample size

This is a pilot trial to collect information about the effect of NAC that will potentially guide a future larger trial, thus there was no formal power calculation. Therefore, the sample size for the proposed trial will be 20-per arm and thus 60 participants will be enrolled for three arms. However, previously we have found a withdrawal/non-evaluable subject rate from our previous TB clinical trials estimated at 10% [33]. Therefore, a total of 66 MDR-TB patients will be recruited for this clinical trial [34].

### Recruitment

The onsite research coordinators will immediately review newly diagnosed or referred MDR-TB patients and administer processes for potential eligibility and assessment. Likewise, communication with physicians referring patients for MDR-TB treatment from different centres will eventually sensitize potential participants interested to participate to receive referral to KIDH. This will facilitate recruitment.

### Assignment of interventions: allocation

#### Sequence generation

**Fig. 2:**
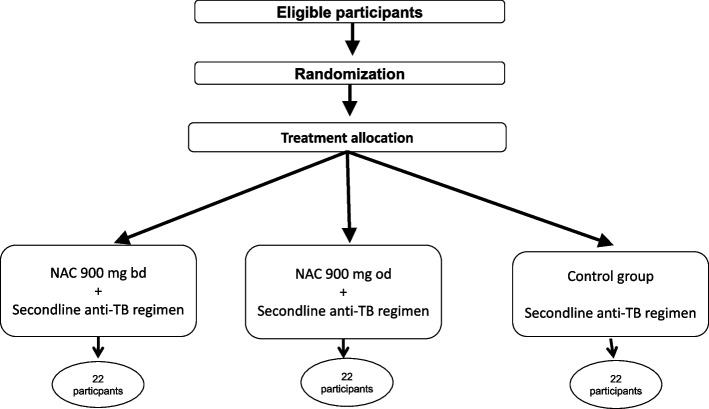
All patients who give consent for participation and those that fulfil the inclusion criteria

All patients who give consent for participation and those that fulfil the inclusion criteria will be randomized to either the control or the experimental groups in 1:1:1 allocation as per a computer-generated random schedule using permuted blocks of randomization (Fig. 2).

### Concealment mechanism

This is an open label study. Both the research participants, the investigator team and health care providers will be aware of the assignment. To prevent selection bias by facilitating enrolment of the comparable participants in each arm, allocation concealment will be ensured. Participants will complete all baseline measurements prior to release of the randomization code. A computer-generated randomization numbers will be printed. For every randomization number a code for a treatment will be assigned.

Primary endpoint (ADRs) will be measured by both clinical assessment and laboratory measurements. To avoid ascertainment bias in the measurement of other endpoints such as adverse events, or performance bias in decision to discontinue or modify treatment, or exclusion/attrition bias in the decision to withdraw from the trial or exclude a participant from analysis, blinding will be implemented. Blinding will be at two levels; the trial participants and health care providers. Health care providers include outcomes assessors (physicians and technicians such as aaudiologists), data collectors, laboratory staff, nurses, and pharmacists.

### Implementation

An allocation sequence will be generated by the sponsor representative pharmacist. Site investigators will enroll participants while the site research pharmacist(s) will assign participants to interventions.

### Assignment of interventions

This is an open label thus research participants and the investigator team and health care providers will be aware of the assignment. The later includes clinicians, and data collectors.

### Data collection and management

All case record forms (CRF) both electronic and hard copy pages will be completed for every participant receiving any amount of IMP. Clinical information will be captured in eCRF while laboratory information will be captured in hard copy CRF. For screening failure participants, a screening failure eCRF will be completed. For subjects who are prematurely withdrawn, the visits up to withdrawal plus the withdrawal and follow-up visits need to be completed.

Source documents are defined as all information in original records and certified copies of original records of clinical findings, observations, or other activities in a clinical trial necessary for the reconstruction and evaluation of the trial. Source documents will include, but are not limited to, progress notes, electronic data, screening logs, and recorded data from automated instruments.

All source documents pertaining to this trial will be maintained by the investigators. Source documents will be available for trial-related monitoring, audits, Institutional Review Board (IRB) review and regulatory inspections providing authorized persons direct access to source documents.

### File management at the trial site

It is the responsibility of the investigators to ensure that the trial centre files are maintained in accordance with International Good Clinical Practice Guidelines and the ethical principles that have their origin in the Declaration of Helsinki.

### Records retentions at the trial site

The investigator will retain records and data from the trial for safety reasons and for audit and inspection subsequent to trial completion. The essential documents will be retained according to ICH-GCP guideline. The sponsor will make financial provisions for the investigator to deposit the documents at an external site for safekeeping for as long as required by regulations and the sponsor.

### Plans to promote participant retention and complete follow-up

Although the site has experience with retaining up to 100% of research participants in longitudinal trials, refresher training of research staff will be conducted to maintain the enthusiasm and perseverance of research staff but also, high morale, and compassion to engage and relate with patients. We have found this an important strategy in achieving high retention rates.

### Data management

CRFs will be filled in a timely, accurate and legible manner. Electronic-CRF will be populated in the database while the laboratory CRF entries will be verifiable to source documentation other than the CRF. The CRFs will be filled electronically, in a timely, accurate and legible manner into the Research Electronic Data Capture (REDCap), a highly secured web-based application to capture data for clinical research. CRF entries will be verifiable to source documentation other than the CRF. Site Standard Operating Procedures will be adhered to for all clinical and bioanalytical activities relevant to the quality of the trial. Subject compliance will be monitored throughout the trial. The investigator will be signing and date any test results to verify that the results have been reviewed. The investigator may appoint other sub-investigators to assist with the trial. However, the lead investigator maintain responsibility for the trial and will supervise the sub-investigators. Written IEC/IRB approval will be obtained prior to involvement in the trial. The investigator will ensure that all site personnel are adequately trained in GCP, the protocol, IB, and all trial procedures and requirements.

The study will be monitored to verify that the rights and well-being of human subjects are protected; that trial data are accurate, complete and verifiable with source data; and that the trial is conducted in compliance with the protocol, International GCP, the ethical principles that have their origin in the Declaration of Helsinki and the applicable regulatory requirements.

Monitors assigned by the sponsor will conduct regular site visits for the purpose of monitoring various aspects of the trial. Visits will take place within a predetermined interval, but this may vary during the course of the trial. The investigator and site staff will allow the trial monitor and authorized representatives of the sponsor to inspect all CRFs, written informed consent documents and corresponding source documents (e.g., original medical records), subject records and laboratory raw data, and (2) access clinical supplies, dispensing and storage areas. The investigator and site staff will also assist with monitoring activities if requested and provide adequate time and space for monitoring visits. The monitor will query any missing, confusing, spurious, or otherwise ambiguous data with the investigator. All queries will be resolved in a timely manner. A monitoring log will be maintained recording each visit, the reason for the visit, the monitor’s signature and investigator or designee’s confirmation signature.

### Confidentiality

All site staff, the sponsor, and any sponsor representatives will preserve the confidentiality of all subjects taking part in the trial, in accordance with International GCP, applicable local legislation/regulations. Subject to the requirement for source data verification by the trial personnel by reference to the subject’s notes, confidentiality of all subject identities will be maintained. Only subject trial number and initials will be used on the CRF and in all trial correspondence, as permitted. No material bearing a subject’s name will be kept on file by the sponsor. The written informed consent will contain a clause granting permission for review of the participants’ source data.

## Statistical methods

### Statistical methods for primary and secondary outcomes

The primary efficacy analysis will be conducted using laboratory measurement. Analysis that will be performed includes both Modified Intent to Treat (MITT) defined as analysis of all randomised participants and a Per Protocol (PP) defined as comparison of three arms; control with either 900 mg OD or 900BD or both intervention arms in participants who completed the treatment originally allocated.

The difference in the proportion of patients with ADRs (as defined by the primary efficacy endpoint) between each treatment arm and control arm will be calculated with 95% confidence interval using standard methods**.** In addition, hearing threshold level will be analysed over time using mixed effect models to account for the longitudinal nature of the data. Mean differences between arms in change from baseline (with 95% confidence intervals) will be derived from the fitted model.

The incidence of severe adverse drug reactions as defined by Common Terminology Criteria for Adverse Event (CTCAE) will be calculated in percentage of affected individuals in each arm during the exposure time period of 6 months and the relative risks will be estimated. Time to stable conversion in both phenotypic and molecular (TB molecular bacterial load assay) MTB monitoring methods will be calculated and compared in each arm using adjusted and unadjusted hazard ratio with log-rank test. Furthermore, the rate of patients in each arm converting to negative sputum culture at 4 or 6 or 8 months after treatment initiation will be estimated. Kaplan-Meir estimator will be used to estimate the proportion of patients converting to negative culture and MBLA by specific time.

### Pharmacokinetics analysis

Plasma concentrations will be used to build a population PK models to evaluate the effects of NAC on the distribution, metabolism, and excretion of MDR-TB drugs.

### Mycobacterial characterization

Descriptive summary statistics of *M. tuberculosis* isolate susceptibility will be presented.

### Interim analyses

There will be no interim analysis, apart from approximately 6-monthly safety review by the DSMB (see below). The final analysis will be performed when the last participant has completed the last trial procedure. There will be database lock, data analysis, and trial reports generated from this trial.

### Methods in analysis to handle protocol non-adherence and any statistical methods to handle missing data

We will report clearly any missing data and deploy multiple imputations under the missing at random assumptions for the missing outcomes or covariates, which we anticipate to minimal and not systematic.

### Plans to give access to the full protocol, participant level-data and statistical code

Data will be available and will be accessed in appropriate data management portals, yet personal information that will identify the participants will not be shared.

## Oversight and monitoring

### Composition of the coordinating center and trial steering committee

There will be a Clinical Trial Management Group (CTMG) which will undertake all sponsorship responsibilities to ensure that the conduct of the clinical trial comply with Medicines for Human use Regulations of 2004 and subsequent amendments for regulated trials. Five members of the CTMG are composed with the sponsor-investigator, the co-investigator also serves as pharmacotherapy expert, the clinical microbiologist, study doctor also a PhD trainee on clinical trials, and quality assurance largely focusing on safety who has a decade experience of working in clinical trials but also attached for one year at Novartis Pharma under the EDCTP Clinical Research and Development Fellowship. The CTMG will ensure the right, safety, dignity, and well-being of the participants are protected and take priority over other interests while the data generated are reliable and robust.

### Composition of data safety monitoring board (DSMB), its role and reporting structure

A DSMB with three members will be appointed with a primary responsibility of an act in an advisory capacity to the sponsor to safeguard the interests of trial subjects by monitoring subject safety, assess subject risk versus benefit, assess data quality and general evaluation of the trial progress. DSMB activities will be delineated in a term of agreement that will define the membership, responsibilities and the scope and frequency of data reviews. The DSMB will operate on a conflict-free basis independently of the sponsor and the trial team. It will comprise 3 voting members. The DSMB will have an organisational meeting prior to commencement of the trial. The DSMB will meet approximately every six months and at least annually when it will review unblinded data during a closed session. The sponsor or the DSMB may convene ad hoc meetings if safety concerns arise during the trial. After its assessment, the DSMB will recommend to the sponsor continuation, modification or termination of the clinical trial.

### Adverse event reporting and harms

Adverse events will be collected by the investigator from the time a subject sign the Informed Consent Form through to their end of follow-up visit. Participants that will be early withdrawn, will only have SAEs collected at the time of withdrawal. Any AE (serious or non-serious) observed by the investigator or reported by the subject will be recorded on the Adverse Event Case Report Form. The investigator will review each AE and assess its severity and relationship to drug treatment based on all available information at the time of the completion of the case report form. AE with relationship with drug treatment will be termed as ADRs and severity grading will be according to the CTCAE.

In the case where an overall diagnosis cannot be made, each specific sign and/or symptom will be recorded as individual AEs. Documentation of the date of onset, and stop date (duration) if applicable will be done. The AEs will also be described in severity, and action taken with IMP while concurrently describing the action taken to the participant. The outcome and relationship to IMP will be recited. Also, the occurrence and seriousness of the AEs will be documented.

### Plans for communicating important protocol amendments to relevant parties

Any change to the protocol will be completed by means of a protocol amendment. Any changes, which affect subject safety or welfare, will be submitted to the IRB and Regulatory Authorities prior to implementation. Protocol amendments will be incorporated into the public Partnership for Access to Clinical Trial Registry (PACTR202007736854169).

### Dissemination plans

Results of this research will be submitted for publication as soon as feasible upon completion of the trial. An integrative knowledge translation strategy will be used for involving policy makers at the Ministry of Health and Tuberculosis and Leprosy Programme in Tanzania. Findings of this study will be presented to different audiences including the community through community advisory board meetings, media coverage, and policy briefs. Findings will also be shared at relevant international conferences.

## Discussion

This trial is part of the clinical trial capacity development in Tanzania. To our knowledge, this is the first registered trial to examine the protective effect of NAC on occurrence of ADRs in MDR-TB patients treated with current WHO recommended regimens that prioritize bedaquiline, fluoroquinolones, linezolid, and clofazimine. While this new prioritization removes some renal and hearing loss potential if the injectable aminoglycoside is withheld, there remains considerable risk for hepatoxicity from the combination of bedaquiline, clofazimine and a fluoroquinolone, as well as linezolid related cytopenias and neuropathy. Severe ADRs are independently morbid, but resulting treatment interruptions can also amplify *M. tuberculosis* drug-resistance which is particular concern for loss of efficacy of the novel drugs such as bedaquiline [35]. NAC has been recommended as an adjuvant therapy in certain TB and non-TB settings but currently few programmes have adopted this approach in part due to lack of evidence. NAC trials conducted in DS-TB showed not only a protective effect in DILI but also a delayed onset and hastened resolution of DILI if it did occur [36]. Furthermore, a large cohort study revealed that patients that received pulmonary TB treatment and NAC had a substantial reduction in 90-day all-cause mortality compared to those that did not receive NAC [37].

While NAC may provide an opportunity of reducing severe ADRs, it will be important to determine if NAC causes any adverse events independent of the MDR-TB regimen. A pooled analysis of 83 studies (*N* = 9988) that did not include people treated for MDR-TB described the safety of administration of NAC for > 6 weeks. Important adverse events included the gastrointestinal intolerance such as nausea, vomiting, abdominal pain which increased 1.4–2.2 times compared to those that did not get NAC [22]. Given this potential, and the fact that NAC has not been trialed in within a patient population on current WHO recommended medication regimens for MDR-TB, there is clinical equipoise for our proposed trial design.

The maximum tolerated dose in phase I study that enrolled participants with a mutation associated degenerative cell disease conducted in US population was found 1800 mg twice daily [38]. Considering less body weight and size of the Tanzanian population, we selected two different doses of NAC, 900 mg and 1800 mg daily for this clinical trial. It is clearly shown that increasing the dose of NAC increases the bioavailability and maximal plasma concentration, yet in overdose scenarios hemolysis, status epilepticus, cerebral edema, and death have occurred, thus warranting vigilance [39–41].

### Trial status

Participant recruitment is in progress.

## Supplementary Information

**Additional file 1. **

## Abbreviations

ADR: Adverse drug reaction
AE: Adverse event
ALT: Alanine transaminase
AST: Aspartate transaminase
AUC: Area under the curve
CTCAE: Common Terminologies Criteria for Adverse Events
CTMG: Clinical Trial Monitoring Group
DILI: Drug-induced liver injury
DSMB: Data Safety Monitoring Board
EDCTP: European and Developing Countries Clinical Trials Partnerships
GCP: Good Clinical Practice
GSH: Glutathione
ICF: Informed Consent Form
ICH: International Conference of Harmonization
ICIDR: International Consortium for Infectious Diseases Research
KIDH: Kibong’oto Infectious Diseases Hospital
MDRTB: Multidrug-resistant tuberculosis
MITT: Modified intention to treat
MTB: *Mycobacterium tuberculosis*
NAC: N acetylcysteine
PACTR: Pan-African Clinical Trial Registry
PanACEA: Pan-African Consortium for Evaluating Anti-TB Antibiotics
PID: Patients identification
PK: Pharmacokinetics
PP: Per-protocol
REDCap: Research Electronic Data Capture
SADR: Severe adverse drug reaction
TB: Tuberculosis
WHO: World Health Organization
