# Supplementary material for: Protocol for a feasibility randomized controlled trial to evaluate the efficacy, safety and tolerability of N-acetylcysteine in reducing adverse drug reactions among adults treated for multidrug-resistant tuberculosis in Tanzania
Source: Pilot Feasibility Stud. 2023 Apr 1;9:55. doi: 10.1186/s40814-023-01281-7 (PMC10066962; doi:10.1186/s40814-023-01281-7)
Supplement: Supplementary file 1 — Additional file 1. [file 40814_2023_1281_MOESM1_ESM.pdf]

TB Symptoms Profile

TB symptoms	
Cough	☐ Yes ☐ No
Haemoptysis	☐ Yes ☐ No
Chest pain	☐ Yes ☐ No
Excessive sweating	☐ Yes ☐ No
Weight loss	☐ Yes ☐ No
Fever	☐ Yes ☐ No
Loss of appetite	☐ Yes ☐ No
